# Vulnerability, social value and the equitable sharing of benefits from research: beyond the placebo and access debates

**DOI:** 10.3389/fmed.2024.1432267

**Published:** 2024-09-17

**Authors:** Chieko Kurihara, Dirceu Greco, Ames Dhai, Kotone Matsuyama, Varvara Baroutsou

**Affiliations:** ^1^Kanagawa Dental University, Yokosuka, Japan; ^2^Ethics Working Group of International Federation of Associations of Pharmaceutical Physicians and Pharmaceutical Medicine (IFAPP), Woerden, Netherlands; ^3^School of Medicine, Federal University of Minas Gerais, Belo Horizonte, Brazil; ^4^School of Clinical Medicine, University of the Witwatersrand, Johannesburg, South Africa; ^5^Department of Health Policy and Management, Nippon Medical School, Tokyo, Japan; ^6^International Federation of Associations of Pharmaceutical Physicians and Pharmaceutical Medicine (IFAPP) President, Woerden, Netherlands

**Keywords:** vulnerability, social value, post-trial access, global health, Declaration of Helsinki

## Abstract

The vulnerability of research participants is a critical topic for the 2024 revision of the Declaration of Helsinki, with the proposal to include “social value. ” However, this proposal has been withdrawn and the relationship between the two concepts has not been clarified. This paper attempts to clarify: (1) the recent reform for the ethical inclusion of vulnerable study participants to promote diversity; (2) the social value, prerequisite for everyone, especially for those who are vulnerable and the most in need; (3) the requirements for promoting the inclusion of vulnerable participants, in particular the review of the norms for placebo-controlled trials and post-trial access; (4) finally, the direction of research ethics reform to achieve social value and equitable global health.

## 1 Introduction

The World Medical Association's (WMA) Declaration of Helsinki (DoH), last amended in 2013 (1) is due to be next amended in October 2024, after an interval of 10 years. In the proposed revision by the WMA the term “social value” was defined as “*the ultimate goal of research involving humans*” in an earlier version, but this was deleted in the second version that went out for public consultation (draft DoH 2024), despite its inclusion being in line with the 2016 version of the CIOMS Guidelines for Health Research (2) (CIOMS 2016). Social value must be achieved through viable strategies of “benefit sharing” (3) and “post-trial access” (4–6) for any research being conducted in host communities. This is in particular to prevent exploitation of resource-poor communities. The debate has been notably contentious in relation to the ethics of placebo-controlled trials (7–9), conducted in vulnerable communities without ensuring the best-proven intervention already in place. This has been criticized as a double-standard (10) and ethics dumping between developed and developing countries.

Diversity of research participants including vulnerable individuals and communities is one of the critical challenges (11–13) in achieving equitable global health. In pursuit of the latter, a true end of research (14), the process must be equipped with mechanisms to avoid any form of exploitation, including the trajectory to go beyond the debates on placebo-controlled trials (9) and post-trial access (4).

Through a narrative, non-systematic literature review, including a comparative analysis between the DoH and CIOMS 2016, and non-systematic discussions among authors as well as various relevant stakeholders (see Acknowledgment and Supplementary information 1), this policy paper attempts to conceptualize the normative implications of protecting vulnerable research participants and ensuring social value. It then discusses in detail the norm of post-trial access and the value of protection effected through community engagement as strategies to achieve this. The authors' proposed revisions to the current DoH in relation to the issues discussed here are presented in the Supplementary information 2. Consistency between the CIOMS 2016 and the draft DoH 2024 is particularly important because the CIOMS guidelines were developed to apply DoH principles to low-resource settings (2). Furthermore, protecting vulnerable populations and respecting the social value in these communities is paramount to achieving global health. The fact that the working groups that developed the CIOMS guidelines included non-physician experts, in contrast to the DoH revisions where the working groups are composed entirely of physicians with non-physician advisors, may also have influenced the nature of both documents.

## 2 Vulnerability

### 2.1 From categorical to contextual vulnerability

The long history of profound ethical concerns for vulnerability has produced several contesting propositions. “Categorical vulnerability” (15) entitles special additional protection to those with weak autonomy or at risk of physical harm [e.g., children, incapacitated adults, women (socially and/or physically vulnerable, including women of childbearing age, pregnant and nursing mothers), prisoners, those at the bottom of the hierarchy, the economically disadvantaged, the stigmatized] (16–18). Vulnerability is one of the characteristics of human beings, that is particularly respected together with autonomy, dignity and integrity (19–22).

At the same time, attention has been drawn to the fact that the level of vulnerability varies according to the context in which an individual or a group is situated. “Contextual vulnerability,” recognizes that everyone can be vulnerable in different situations. This approach will provide appropriately enhanced protection taking into account each situation. CIOMS 2016 provides detailed categorization and possible associated risks, expressing a position that also supports contextual vulnerability.

It must be also noted that all participants in research are by definition vulnerable. This is due not only to the asymmetry of knowledge between the researcher and the research participants as subjects, but also in the case of patients due to the inherent vulnerability associated with the disease and the perceived expectation of benefit from participation in the research.

### 2.2 From exclusive to inclusive protection

Paradoxically, the principle of enhanced protection has been used to exclude these groups from participation in research. Another challenge is “exclusive protection” vs. “inclusive protection.”

Traditionally, vulnerable individuals or groups were excluded from research a priori, and exceptional inclusion needed to be justified. One of the triggers for his trend were the United States (US) regulations that were introduced in the late 1970s and 1980s (15–17), and which required additional protection and limitation of the expected risk. Accordingly, due to exclusion, vulnerable individuals and groups were made even more vulnerable.

CIOMS 2016 was the first international agreement to reverse this notion: vulnerable populations should NOT be excluded from research unless there is sufficient justification (23). This concept was previously adopted by the US National Institutes of Health in 1998 for research involving children. (24) The theoretical reform in CIOMS 2016 is because diverse populations who have been routinely excluded have unique physiologies and health needs, including social perspectives, that have not been well studied. It has been recognized that such situations have led to a lack of evidence for their care. Strong scientific arguments have been put forward to promote the inclusion of diverse underserved groups (12, 25), such as children (26, 27), especially children in minority groups (28), women, especially pregnant women (29), the elderly (30), prisoners (31), the economically disadvantaged (32), including urban slum dwellers (33), indigenous groups (34), together with the strengthening of safeguards. In particular, it should be noted that the promotion of the inclusion of low-and middle- income countries in global studies has not yielded adequate post-trial benefits (35–37).

### 2.3 Benefit sharing as mandatory prerequisites

This trend has been highlighted by experiences such as participation in vaccine trials during pandemics or patients seeking access to promising experimental treatments in disaster settings including in times of wars or conflicts (38, 39). However, it has not eliminated the risk of vulnerable individuals or groups being exposed to risk of research without benefiting from the results. For this emerging safeguard to be defensible, it is essential to ensure the right to post-trial access and sharing of research benefits.

The UNESCO Declaration on Bioethics and Human Rights (21) provides a list of benefit-sharing measures that are not limited to post-trial access. There may be some cases where post-trial access is not relevant, e.g., exploratory or observational studies. However, this list should not be used to select compromised measures where post-trial access is ethically required (40). The principle of the benefit sharing has been agreed to as a global standard in the Convention on Biological Diversity (41, 42) to avoid exploitation of the communities holding biological resources, and to pursue benefit-sharing by all means including technology transfer and capacity building.

Only on this premise, could vulnerable individuals supported by advocates realize their full potential, through learning by pedagogy offered to even the most oppressed (43). According to Freire's Pedagogy of the Oppressed (43), “emancipation” in the sense of “bottom-up” participation by disadvantaged people is critically more important than “empowerment” meaning “top-down” education and inclusion by the experts. Through such a process of collaboration, vulnerable research participants can understand that their altruism could truly benefit others (44, 45). In addition to protecting the risks of individual research participants, mechanisms must be put in place to ensure that the benefits from research are available and affordable to these individuals and those in need in the global community.

## 3 Social value

### 3.1 Scientific and social values

Philosophy of science in the late 20th century raised debates on the distinction between cognitive value of science generating knowledge and non-cognitive (including social) value of science, considering the consequences of application of this knowledge (46). Debates on utility and safety in the application of science led to the emphasis on social responsibility of scientists (47).

In the context of health-related science involving humans, “social value” was implied in the “justice” principle (48), one of the well-established three principles of research involving humans along with “respect for autonomy” and “beneficence” (16). The “beneficence” principle requires justification of risk to study participants, which are to be minimized and to be outweighed by prospective direct benefit to the individual in addition to the scientific value of generating knowledge. The Belmont Report states that “*the risks and benefits affecting the immediate research subject will normally carry special weight*.” The “justice” principle requires avoidance of such exploitation where vulnerable individuals or groups are more exposed to risks of research without sharing benefit from research. This suggests that scientific knowledge from research must benefit study participants and their community, which means social value for them.

Similar terminology such as “public value” is used sometimes with similar implications (49) to “social value.” However, historical development of the “public value” (50, 51) suggests that the implication of “public” is not limited to specific types of groups of people, and in the context of health research, tends to be related to data-driven research (49, 52). Meanwhile, “social value” in the context of health research has been discussed to avoid exploitation of specific vulnerable groups of people, especially in the case of interventional research in resource-limited settings. Thus, “*relevance to significant health problems*” (2) of specific target groups is strictly questioned (53). “Social value” is also different from “patient value” which has been discussed in the context of patient-centered medicine (54) or value-based healthcare (55). This paper focuses only on social value as has been discussed in the two international documents, i.e., DoH and CIOMS on research involving humans especially related to vulnerability.

### 3.2 Social value as a necessary condition

Through the debates on placebo-controlled trials, “social value” was proposed as one of the principles, especially for collaborative health research with developing countries (56). CIOMS 2016 was the first international instrument that adopted this principle, describing social and scientific value as “*the fundamental justification for conducting research.”* It defines scientific value as “*a necessary but not a sufficient condition*” required for achieving social value. “Social value” in CIOMS 2016 was reinforced by another guideline on “community engagement,” regarding science as co-creation with the relevant community (57).

In contrast, the first publicly available draft of the revision of the DoH described social value as the ultimate goal, suggesting that the researcher should aim to generate scientifically valid results that lead to future development in conjunction with social value, even if this is not achievable within an individual research protocol. However, this was deleted in the subsequent draft because during the public consultation there were opinions expressing “*concern with the vagueness*.”

This difference may be partially due to the fact that the CIOMS 2016 analyses the justification of research that is recognized by different stakeholders, whereas the DoH mainly discusses the responsibility of an individual physician dealing with an individual patient (48), hence trying to justify the difficulty of achieving social value in an individual research protocol.

### 3.3 Re-conceptualization of “social value”

In summary, CIOMS 2016 guidelines (not limited to the Guideline 1), provide for “social value,” which could be achieved by identifying the research question and design that responds to the health needs of the community in which the research is conducted. Moreover, through stakeholders engagement, there needs to be sufficient expectations to ensure the results of the research are available and affordable to that community.

CIOMS 2016 and other related literatures do not sufficiently provide the philosophical basis for this notion in relation to vulnerability. In this paper the authors argue that this notion of “social value” could be supported by a communitarian interpretation of the Rawls 1971 “Theory of Justice” (58) to prioritize the most vulnerable. Rawls argued that individuals who are free of values in the “veil of ignorance” choose policies that benefit the most vulnerable so that they can benefit even when they are disadvantaged. Communitarians criticized such individualistic thinking, capturing human traits of living in communities and influencing each other. In the context of research ethics, the theory of justice to prioritize the most vulnerable could be interpreted as strengthened by communitarianism, with respect for the values of the relevant community to which each individual belongs. Afro-communitarianism, in particular, functions by sharing values of humanity based on justice, reciprocity and solidarity, and advocates a theory that emphasizes the social values shared by the global community, as argued considering the COVID-19 experience (59).

## 4 Mechanism to ensure post-trial access

### 4.1 Downgrading in the revisions of the DoH

The norm of post-trial access (PTA, hereafter) was first introduced in the 2000 revision of the DoH, which stated that “*At the conclusion of the study, every patient entered into the study should be assured of access to the best proven prophylactic, diagnostic and therapeutic methods identified by the study*.” Later in 2004, a “note of clarification” developed by a small working group was added, suggesting that “*Post-trial access arrangements or other care must be described in the study protocol so the ethical review committee may consider such arrangements during its review.”* Along with the subsequent revisions, this standard was downgraded as one of the items in the study protocol to be assessed by the Research Ethics Committee (REC) and in the informed consent process to leave it to the choice of study participants (4).

One of the reasons for this downgrading is that the sponsor companies sophisticatedly argued that it is impossible to guarantee access immediately after the completion of trial because of the time lag in obtaining regulatory approval for the proven intervention. Another reason from the side of the WMA is its position that PTA is an obligation of the state, and not of an individual researcher (60). This led to the rejection of the DoH in Latin American countries (9, 61), while in Brazil there is a legal obligation to implement what was proposed by the 2000 version of the DoH (4, 40).

In the draft DoH 2024, collaborative arrangements for providing access between relevant stakeholders have been strengthened, but it is a critical issue that this paragraph specifically provides that “*exceptions to these provisions must be approved by a research ethics committee*.” Furthermore, it is not required to be included in the written informed consent document, and participants may not be able to exercise their rights to post-trial access at the end of the trial.

### 4.2 Clarification of responsibility of each stakeholder

There is no doubt that access to safe and effective treatment in public health system is the responsibility of the state to ensure standard of care (SOC) management to its citizens and residents. However, in research the physician-researcher cannot get away from this obligation. This duty is derived from the WMA Declaration of Geneva (62) and the International Code of Medical Ethics (63), cited in the DoH, the former of which derives from the Hippocratic Oath (64). This duty is described in CIOMS 2016 as caring for the health needs of participants (Guideline 6) (2).

The DoH, as an aspirational document (14), needs to clearly state the responsibility of each stakeholder to ensure PTA. PTA of participating individuals and communities is the responsibility of the sponsor, while the state works to include the intervention into its standard of care (SOC). Researchers, civil society and state representatives should engage in advocacy activities toward including the intervention that was shown to be safe and effective in the SOC. If the intervention cannot be included in the SOC, it raises questions about the social value of the trial and whether the trial should have been conducted in that location in the first place (65, 66). RECs need to be well aware of this when reviewing the studies. To ensure the PTA, the mandatory prerequisite and obligations of each stakeholder must be clarified:

#### 4.2.1 Sponsor

When a pharmaceutical company sponsors a clinical trial, the primary responsibility for the PTA lies with the sponsor company. Many countries have guidelines that allow access in the interval between the end of individual participation and regulatory approval, through a study extension, conducting new safety studies, as well as expanded access programs. PTAs are sometimes used to justify “seeding trials” (67, 68) for marketing purposes, the aim of which is “*to influence clinicians who participate in the study to prescribe a new medication rather than to produce knowledge about the merits of these interventions*” (2). This is just one example in CIOMS 2016 of research that lacks social value. Therefore, it is imperative to reinforce that it is corporate social responsibility in terms of business ethics to guarantee the organizational mechanism to ensure PTA for those who need it around the world. This includes establishing a corporate philosophy that prioritizes public health over the commercial benefits of patent-protected products (69). Including this notion in Corporate Social Responsibility (CSR) will contribute to achieving the Sustainable Development Goals (SDGs) (70) through technology transfer and strengthening of local manufacturing capacity (5, 6).

#### 4.2.2 State and health authorities

If the investigational products are found to be safe and effective, the gap to regulatory approval should be bridged by an extension studies, a safety study or an expanded access programe, and such a system should be adequately established by the state to ensure the patient safety and data integrity. The state has a responsibility to provide access for its citizens, not just to study participants. This decision will need to be made in coordination with a health technology assessment (HTA) process, enabling cost-effectiveness assessment. HTA including from the perspective of global health and not limited to the domestic perspective is a critical challenge (71). Access will be facilitated through price negotiations with effective participation of national, regional, and/or international health authorities and also through the necessary revision or waiver (72) of some items of the World Trade Organization's Trade-Related Aspects of Intellectual Property Rights Agreement on drugs and medical products. The certainty that health-related products and the supporting data for safety and efficacy of these products are public goods and not a commodity on the market must be the cornerstone of global public health policies.

#### 4.2.3 Physician researcher

Essentially, the critical responsibility of the physician-researcher is to provide individual participants with the best proven intervention that the participants need after the completion (including termination) of the study as suggested in the above-mentioned Guideline 6 of CIOMS 2016 (2). This applies whether the participant is a patient or a healthy volunteer [there has been a case where the discontinuation of a central nervous system study drug may have led the suicide of a healthy volunteer (73, 74)]. If the best care for the individual patient is supposed to include an intervention that has been proven to be safe and effective in the trial, it is the responsibility of the physician-researcher to request the sponsor to ensure this access at the time of clinical trial planning.

There would be some barriers leading sponsors who want to reduce clinical trial costs to express concerns that development costs would affect drug costs. However, there have been cases where sponsors have willingly changed their trial protocol in response to medical ethics requirements, suggesting, for example, that the clinical trial costs are not as high relative to marketing costs (75).

#### 4.2.4 Community engagement (patient and public involvement)

The current DoH mentions responsibility for PTA in relation to sponsors, researchers and host country governments. To this end, the participation of related communities, including non-governmental organizations, and the involvement of well-educated patients and civil society are crucial. Community engagement is well described in the CIOMS 2016, but needs further elaboration, not only to achieve product availability in the local community, but also to address global common health issues.

The newly added article in the draft DoH 2024 requiring a focus on “structural inequalities” in the research context is valuable. However, the associated article on community engagement is weak, as it does not address the “implementation” of research outcomes to ensure post-trial access. Community engagement in relation to research outcomes should not be limited to, as stated in the draft DoH, “understanding and disseminating” of the results. It is paramount that community is engaged in “implementation” of the study outcomes.

## 5 Discussion: global ethics and global health governance

Ensuring the protection of vulnerability and diversity of study participants will lead to equitable global health. Therefore, benefit sharing and PTA are prerequisites that must be included in the norms and standards of global bioethics (76) and ethical global health governance (77, 78), in the context of research involving humans. Its inclusion in the DoH could decisively strengthen and implement this. The DoH is the best-known ethical standard for research involving humans, and it influences government regulations in most countries. However, there are some limitations as it is a medical code of ethics. The proposed DoH 2024 could be an extremely important driving force for achieving the highest ethical standard for research involving humans, which could be implemented by all relevant stakeholders, not just physicians, but also patients, study participants and the public, industry, governments, relevant organizations, equipped with appropriate mechanisms to ensure the right to health for everyone in the global community.

The authors of this paper are directly and indirectly involved in the debate on the 2024 revision of the DoH, and the CIOMS 2016. However, the incorporation of our ideas into the latest draft of the DoH has been limited, as the issues discussed here have always raised contested arguments, along with conflicting opinions. We will continue to bring our arguments to the rest of the WMA's review process as well as to the collaborative work of disseminating a new 2024 version, and even if not all of them are incorporated, we believe that the discussions on these topics would improve the actual research practice. In addition, certain relevant stakeholders including health-related governmental, non-governmental organizations and regulatory authorities, to which we belong or are affiliated, have already incorporated or will incorporate the standards we advocate. For this reason, the DoH is an important driver of the global community's aspirational commitment to the pursuit of the highest ethical standards, without compromising scientific rigor and integrity of research involving humans.

## 6 Conclusion

To finally achieve social value and equitable global health, the revised DoH must include the following clear principles:

- “Social value” as “the ultimate goal of research involving humans,” as it was in the first draft proposed by the WMA and also, as stated in CIOMS 2016, “the fundamental justification for conducting research,” which scientific value alone cannot satisfy.
- Reinforce the fundamental policy of inclusion of vulnerable individuals or groups in research and the justification of the need for exceptional exclusion, provided that sufficient and rigorously reinforced safeguards and benefit sharing are guaranteed.
- Post-trial access must be guaranteed to study participants, the host community and, ultimately, to those who are most in need in the global community. This leads to “social value” which is the ultimate goal of research, involving diverse study populations, including the most vulnerable individuals and groups.
