# Supplementary material for: Vulnerability, social value and the equitable sharing of benefits from research: beyond the placebo and access debates
Source: Front Med (Lausanne). 2024 Sep 17;11:1432267. doi: 10.3389/fmed.2024.1432267 (PMC11442373; doi:10.3389/fmed.2024.1432267)
Supplement: Supplementary file 1 [file Table_1.pdf]

Supplementary Material-1

Table 1

A methodological aspect of the manuscript to have non-systematic discussions, in addition to non-systematic literature review, without a specific intention of reporting research result, but with explicit intention of improving the 2024 revision of the Declaration of Helsinki. (Information is limited to the cases when authors took the role of organizers. The cases that authors are invited by other organization(s) including the WMA are not mentioned.)

**Name or specification of organizations in which non-systematic discussions specific to the issue of the 2024 revision of the Declaration of Helsinki**	**Style and volume of discussions; involvement of the author(s) of this manuscript; URLs of related information**
**Monthly discussions**	
A voluntary working group of patient and public in collaboration with Japanese Institute for Public Engagement (Ji4pe). (Renamed since July 2024 as Bioethics Working Group of the Ji4pe.)	Monthly web meetings since 2020; CK used to be one of the leaders (until July 2024), taking a role of editorial work of opinions raised from the members whose position is patients and/or public. CK and KM have been involved in some educational projects of Ji4pe. http://cont.o.oo7.jp/grareco.html
Ethics Working Group of the International Federation of Associations of Pharmaceutical Physicians and Pharmaceutical Medicine (IFAPP)	Monthly web meetings to discuss the revision of the DoH (meeting agenda include other topics) since 2018. CK has led this topic since the beginning and KM has been the chairperson of this working group since June 2021. VB has been elected the President of IFAPP in October 2022 and a member of Ethics Working Group of IFAPP since September 2018.
Clinical Development Working Group of the Japanese Association of Pharmaceutical Medicine (JAPhMed, Japanese member association of IFAPP)	Nearly monthly web meetings to discuss about the revision of the DoH since February 2024. CK has been taking a role of making presentations and raising discussions.
128th Pharmaceutical Study Group meeting	A one-time web meeting where Japanese experts and non-experts attended. CK, an organizer of this group, took a role of lecturer to introduce the first public consultation version of the revision of the DoH.
**Webinars and publications**	
Webinar titled "The 2024 Declaration of Helsinki: Taking Forward Bioethics and Human Rights." (5 and 26, August, 2024)<br>(Proceedings planned to be published.)	All the authors were involved in the development of the project plan and contributors to the webinar. CK, DG, KM and VB are members of the organizing and main supporting organizations (Brazilian Society of Bioethics, IFAPP, Japan Association for Bioethics, and others).<br>http://cont.o.oo7.jp/sympo/eigh.html
Publication of a book titled "Ethical innovation for global health: pandemic, democracy and ethics in research" (Springer; November 2023) and two days webinar to disseminate information of publication (December, 2023)	CK, DG and AD are the editors, and VB and KM are chapter authors of this book and made presentations at the two days webinar. Authors and webinar panelists include representatives from the WMA (Secretary General of the WMA and the DoH 2013 revision workgroup chair) and CIOMS (Secretary General and technical editor).<br>https://link.springer.com/book/10.1007/978-981-99-6163-4<br>(Webinar information)<br>http://cont.o.oo7.jp/sympo/eigh.html

Webinar titled "COVID-19 and bioethics Part 3: Pandemic and research ethics: Democracy, placebo and post-trial access" (June 2021) and publication of its proceedings (December 2021).	CK, DG were the organizers, AD was a panelist of the webinar, and the three of them were contributors to the proceedings published in Clinical Evaluation, Vol. 49, Sup. 38. The Secretary General of the WMA and the DoH 2013 revision workgroup chair participated in both of webinar and proceedings. http://cont.o.oo7.jp/49sup38/49sup38contents_e.html
Presidential Symposium in the 40th Annual Scientific Meeting of the Japanese Society of Clinical Pharmacology and Therapeutics, 2019, titled "International Collaborative Research and New Trends of Research Ethics" and publication of its proceedings (June 2020).	DG and Secretary General of the WMA were invited lecturers, CK was one of the organizers of the symposium. The three of them are contributors of the proceedings, published in Clinical Evaluation, Vol. 48, No. 1. http://cont.o.oo7.jp/48_1/48_1contents_e.html
**Webinars by IFAPP under the MoU with the WMA and publications in IFAPP TODAY**	
Workshop titled "IFAPP Workshop on the future revision of the Declaration of Helsinki: Dialogue with the WMA" in the IFAPP's annual meeting (ICPM) (October 2022)	WMA workgroup chair of the revision of the DoH and Secretary General of the WMA were invited and CK was an organisor/moderator. The webinar report was published in IFAPP TODAY. 2022; Nov/Dec (29)：5-8.
Workshop titled "IFAPP & WMA Workshop on the revision of the Declaration of Helsinki focusing on Data-Driven Research" in the IFAPP's European regional meeting (June 2023)	WMA workgroup member and advisor of the revision of the DoH were invited and CK and KM were organisor/moderator. The webinar report was published in IFAPP TODAY. 2023; May (34):8-12.
