# Supplementary material for: Vulnerability, social value and the equitable sharing of benefits from research: beyond the placebo and access debates
Source: Front Med (Lausanne). 2024 Sep 17;11:1432267. doi: 10.3389/fmed.2024.1432267 (PMC11442373; doi:10.3389/fmed.2024.1432267)
Supplement: Supplementary file 2 [file Table_2.pdf]

frontiers

Supplementary Material-2

Table 2

Authors' proposed revision to the Declaration of Helsinki (DoH), for transforming from categorical to contextual vulnerability associated with reforming from exclusive to inclusive protection. (Categorical/contextual vulnerability and exclusive/inclusive protection are discussed in the main text. Other necessary revisions not directly related to the topic are not necessarily included.)

Subheadings and paragraphs (Para.) of 2013 version of the DoH. **Bold, underlined** texts indicate the need for change.	Authors proposed changes (**Bold, underlined, or deleted**) related to categorical/contextual vulnerability and exclusive/inclusive protection.
Rationale of the needs for changes (texts in *italic* are quoted from original)	
General principles	(No need to change the term.)
(Para. 1 to 15 present general principles among which Para. 13 is related to vulnerability.)	
6. The primary purpose of medical research involving human **subjects is** to understand the causes, development and effects of diseases and improve preventive, diagnostic and therapeutic interventions (methods, procedures and treatments).	**7**. The primary purpose**s** of medical research involving human **participants are** to understand the causes, development and effects of diseases, and improve preventive, diagnostic and therapeutic interventions ~~(methods, procedures and treatments)~~**, and ultimately provide social value to the advancement of public and individual health.**
13. Groups that are underrepresented in medical research should be provided appropriate access to participation in research.	(No need to change the principle.)
**Vulnerable** Groups and Individuals	**Vulnerability of** Groups and Individuals
19. **Some** groups and individuals are **particularly vulnerable and may** have an increased likelihood of being wronged or of incurring additional harm.<br><br>**All vulnerable groups and individuals should receive specifically considered** protection.	19. **All** groups and individuals **may** have an increased likelihood of being wronged or incurring additional harm.<br><br>**The vulnerability of individuals and groups in research must be analised and given special, additional** protection.

<table>
<tr>
<td>20. Medical research with a vulnerable group is only justified if the research is responsive to the health needs or priorities of this group <u>and the research cannot be carried out in a non-vulnerable group</u>. In addition, this group should stand to benefit from the knowledge, practices or interventions that result from the research.</td>
<td>20. Medical research with a vulnerable group is only justified if the research is responsive to the health needs or priorities of this group<s> and the research cannot be carried out in a non-vulnerable group</s>. In addition, this group should stand to benefit from the knowledge, practices or interventions that result from the research.</td>
</tr>
<tr>
<td colspan="2">1. The principle expressed in Para. 13 does not need change because it represents the principle to promote inclusion of “groups that are underrepresented in medical research”, which represents the position of “inclusive protection”, if it is associated with other changes in this table.
2. Subheading “Vulnerable Groups and Individual” represents “categorical vulnerability” (with premise of existence of already defined certain “groups” or “individuals”), thus it need to be changed to “Vulnerability in Groups and Individuals” which represents “contextual vulnerability” (focusing vulnerability possible to emerge in any group or an individual). We support this change discussed at the regional meeting in Johannesburg.
3. First sentence of Para. 19 also suggests existence of “some” already defined vulnerable groups of individuals. Second sentence should be more focused on changing vulnerability and its natures to be scrutinized and protection to be strengthened.
4. In Para. 20, the text “and the research cannot be carried out in a non-vulnerable group” excludes participation of vulnerable groups/individuals in research even if there is expected direct benefit to individual participants, thus, this text should be deleted.
5. We support the following text of CIOMS and the DoH should be in line with this policy. “A traditional approach to vulnerability in research has been to label entire classes of individuals as vulnerable. The account of vulnerability in this Guideline seeks to avoid considering members of entire classes of individuals as vulnerable. However, it is useful to look at the specific characteristics that may render individuals vulnerable, as this can aid in identifying the special protections needed for persons who may have an increased likelihood of being wronged or of incurring additional harm as participants in research.” This is the reason to strengthen categorial scrutiny in the second sentence of Para. 19 while adopting policy of “contextual vulnerability” deleting a text in Para. 20.</td>
</tr>
<tr>
<td>Informed consent</td>
<td>(No need to change the term.)</td>
</tr>
<tr>
<td>(Para. 25 to 27 discuss about informed consent of incapable individuals. Para. 29 is requirement for assent.)</td>
<td>(Para. 25, 27, 29 may need changes but not discussed here in order to focus the topic.)</td>
</tr>
<tr>
<td>28. For a potential research subject who is incapable of giving informed consent, the physician must seek informed consent from the legally authorised representative. These individuals must <u>not</u> be included in a research study that has no likelihood of benefit for them unless it is intended to promote the health of the</td>
<td>28. For a potential research subject who is incapable of giving informed consent, the physician must seek informed consent from the legally authorised representative. These individuals must <s>not</s> be included in a research</td>
</tr>
</table>

<table>
<tr><td>group represented by the potential subject, the research cannot instead be performed with persons capable of providing informed consent, and the research entails only minimal risk and minimal burden.</td><td>study unless there is justification for exclusion.

The study that has no likelihood of benefit for them ~~unless~~ may be justified when

- it is intended to promote the health of the group represented by the potential subject;
- the research cannot instead be ~~performed~~ conducted with persons capable of ~~providing informed~~ consenting, and
- the research entails only minimal risk and minimal burden.</td></tr>
<tr><td>30. Research involving subjects who are physically or mentally incapable of giving consent, ….may be done only if the physical or mental condition that prevents giving informed consent is a necessary characteristic of the research group.</td><td>~~30. Research involving subjects who are physically or mentally incapable of giving consent, ….may be done only if the physical or mental condition that prevents giving informed consent is a necessary characteristic of the research group.~~</td></tr>
<tr><td colspan="2">1. The second sentence of Para. 28 and Para. 30 represent “exclusive protection”, thus the second sentence should be changed to expression of “inclusive protection” and Para. 30 (which excludes participation of incapable individuals in research with expected direct benefit to them) should be deleted.
2. We support the conditions of current DoH to permit research with no individual direct benefit to individual participant incapable of consent: (1) intended to promote health of target group; (2) impossible to be performed instead with capable individuals; and (3) minimal risk/burden. This set of conditions do not need the change but better to be in bullet points.
3. We support the CIOMS to take the position of “inclusive protection” sating that “Adults who are not capable of giving informed consent must be included in health-related research unless a good scientific reason justifies their exclusion.”; also CIOMS’s conditions for research with no direct benefit to incapable adults are, almost same as current DoH, set as follows: (1) “these interventions and procedures target conditions that affect persons who are not capable of giving informed consent as well as those who are capable, unless the necessary data cannot be obtained without participation of persons who are incapable of giving informed consent” AND (2) “the risks must be minimized and no more than minimal.”</td></tr>
<tr><td>Use of placebo</td><td>(No need to change the term.)</td></tr>
<tr><td>33. The benefits, risks, burdens and effectiveness of a new intervention must be tested against those of the best proven intervention(s), except in the following circumstances:

Where no proven intervention exists, the use of placebo, or no intervention, is acceptable; or</td><td>33. The benefits, risks, burdens and effectiveness of a new intervention must be tested against those of the best proven intervention(s) in the world, except in the following circumstances:

Where no proven intervention exists, the use of placebo, or no intervention, is acceptable; or</td></tr>
</table>

<table>
<tr>
<td>Where for compelling and scientifically sound methodological reasons the use of any intervention less effective than the best proven one, the use of placebo, or no intervention is necessary to determine the efficacy or safety of an intervention

and the patients who receive any intervention less effective than the best proven one, placebo, or no intervention will not be subject to additional risks of serious or irreversible harm as a result of not receiving the best proven intervention.

Extreme care must be taken to avoid abuse of this option.</td>
<td>Where for compelling and scientifically sound methodological reasons the use of any intervention less effective than the best proven one, the use of placebo, or no intervention is necessary to determine the efficacy or safety of an intervention

and the patients who receive any intervention less effective than the best proven one, placebo, or no intervention will not be subject to additional risks of **~~serious or irreversible~~** ~~harm~~ as a result of not receiving the best proven intervention**, and clinical equipoise or uncertainty among the comparator arms is confirmed.**

~~Extreme care must be taken to avoid abuse of this option.~~</td>
</tr>
<tr>
<td colspan="2">1. Revision of placebo clause is necessary condition to adopt “inclusive protection” policy in order to avoid exploitation of vulnerable individuals and populations.
2. To avoid misunderstanding and double standard, “the best proven intervention(s)” must be clearly described as it is the best proven “in the world”.
3. Vulnerable individuals, including those who are incapable of consent, may be involved in placebo arm, which is theoretically no expect of individual direct benefit. The current DoH permitting high limit of risk “no *additional risks of serious or irreversible harm*” (permitting moderate harm or substantial burden, which are reversible after long term) is inconsistent with conditions in Para. 28. Any type of research must seek for risk minimization. Also, we should consider CIOMS set this condition of risk: “minor increase above minimal risk”.
4. While the notions “Clinical equipoise” and “uncertainty” have caused debate, such statement that “we don’t know which arm of the compared interventions is the best” is easy to understand for lay member of the REC and candidate participants.
5. “Where for compelling…best proven one(s)” and “*Extreme care … this option*” should be deleted because these are applicable in any type of study.</td>
</tr>
<tr>
<td>Post-trial **provisions**</td>
<td>Post-trial **access and benefit sharing**</td>
</tr>
<tr>
<td>34. In advance of a clinical trial, sponsors, researchers and host country governments should make provisions for post-trial access for all participants who still need an intervention identified as beneficial in the trial. This information must also be disclosed to participants during the informed consent process.</td>
<td>34. In advance of a clinical trial, **the respective responsibilities of** sponsors, researchers and host country governments, and **other relevant stakeholders** should ~~make~~ **be clarified in the** provisions for **ensuring** post-trial access for all participants, **host communities** who **~~still~~** need an intervention identified as beneficial in the trial. This ~~information~~ **provision** **~~must~~** **needs to be developed through stakeholder involvement** ~~also be disclosed to participants~~</td>
</tr>
</table>

<table>
<tr><td></td><td>~~during the~~ **and disclosed to Research Ethics Committee in study protocol and** **to candidate study participants in** informed consent ~~process~~ **form, which will be registered in public database**. **The plan of sharing the benefits with those who most need of the intervention in the world should also be included in the provision.**</td></tr>
<tr><td colspan="2">1. Revision of post-trial access clause is necessary condition to adopt “inclusive protection” policy in order to avoid exploitation of vulnerable individuals and populations.
2. Current DoH requires only items of post-trial provision in protocol and informed consent process, which is downgraded from the 2000 version which established the norm of post-trial access entitled to study participants. In addition, post-trial access must be assured to the host community as well as those who most in need in the world. This can be achieved through stakeholder involvement strategy clarifying responsibility and role of each, especially, sponsor, government and researcher.
3. “Informed consent process” suggests that it can be only oral explanation, but it should be in written informed consent form (ICF). Also, this information should be included in items registered in public database.
4. Brazilian Research Ethics Committee Resolution 466/2012* is a good example to implement post-trial access as legally enforceable policy: “*III.2 – Research involving human beings in any area of knowledge must: ensure to the participants adequate conditions of follow up, treatment, access to new drugs (if shown to be safe and effective); III.3 – In biomedical research: d) At the end of the study the sponsors must ensure to all participants, access, free of charge and for all needed time, to the best prophylactic, diagnostic and treatment that have demonstrated to be efficacious; d.1) Access will also be warranted between the end of individual participation and the end of the study. In this specific situation access will be permitted through a study extension, according to a consubstantiated analysis of the participant’s attending physician.*”
* It succeeded Resolution 404/2008 (1 August 2008), including “to propose further discussion on access to health and to the products that have shown efficacy to all who need them.</td></tr>
</table>
